# Dose Prediction Using a Three-Dimensional Convolutional Neural Network for Nasopharyngeal Carcinoma With Tomotherapy

**DOI:** 10.3389/fonc.2021.752007

**Published:** 2021-11-11

**Authors:** Yaoying Liu, Zhaocai Chen, Jinyuan Wang, Xiaoshen Wang, Baolin Qu, Lin Ma, Wei Zhao, Gaolong Zhang, Shouping Xu

**Affiliations:** ^1^ Department of Radiation Oncology, the First Medical Center of the People's Liberation Army General Hospital, Beijing, China; ^2^ School of Physics, Beihang University, Beijing, China; ^3^ Manteia Technologies Co., Ltd, Xiamen, China

**Keywords:** dose prediction, deep learning, Tomotherapy, nasopharyngeal carcinoma, radiotherapy plan

## Abstract

**Purpose:**

This study focused on predicting 3D dose distribution at high precision and generated the prediction methods for nasopharyngeal carcinoma patients (NPC) treated with Tomotherapy based on the patient-specific gap between organs at risk (OARs) and planning target volumes (PTVs).

**Methods:**

A convolutional neural network (CNN) is trained using the CT and contour masks as the input and dose distributions as output. The CNN is based on the “3D Dense-U-Net”, which combines the U-Net and the Dense-Net. To evaluate the model, we retrospectively used 124 NPC patients treated with Tomotherapy, in which 96 and 28 patients were randomly split and used for model training and test, respectively. We performed comparison studies using different training matrix shapes and dimensions for the CNN models, i.e., 128 ×128 ×48 (for Model I), 128 ×128 ×16 (for Model II), and 2D Dense U-Net (for Model III). The performance of these models was quantitatively evaluated using clinically relevant metrics and statistical analysis.

**Results:**

We found a more considerable height of the training patch size yields a better model outcome. The study calculated the corresponding errors by comparing the predicted dose with the ground truth. The mean deviations from the mean and maximum doses of PTVs and OARs were 2.42 and 2.93%. Error for the maximum dose of right optic nerves in Model I was 4.87 ± 6.88%, compared with 7.9 ± 6.8% in Model II (*p*=0.08) and 13.85 ± 10.97% in Model III (*p*<0.01); the Model I performed the best. The gamma passing rates of PTV_60_ for 3%/3 mm criteria was 83.6 ± 5.2% in Model I, compared with 75.9 ± 5.5% in Model II (*p*<0.001) and 77.2 ± 7.3% in Model III (*p*<0.01); the Model I also gave the best outcome. The prediction error of D_95_ for PTV_60_ was 0.64 ± 0.68% in Model I, compared with 2.04 ± 1.38% in Model II (*p*<0.01) and 1.05 ± 0.96% in Model III (*p*=0.01); the Model I was also the best one.

**Conclusions:**

It is significant to train the dose prediction model by exploiting deep-learning techniques with various clinical logic concepts. Increasing the height (Y direction) of training patch size can improve the dose prediction accuracy of tiny OARs and the whole body. Our dose prediction network model provides a clinically acceptable result and a training strategy for a dose prediction model. It should be helpful to build automatic Tomotherapy planning.

## Introduction

Radiotherapy (RT) Plan optimization is a time-consuming process in routine clinical practice. It may cost several hours to constrain the dose distribution to meet the optimal clinical criteria. The plan quality, which the total voxel information can guide the RT plan optimization and ensure, depends on the medical dosimetrist or the medical physicist’s clinical experience and skills. It can minimize the uncertainty of the planning outcome due to different planners handling the planning process (1–3).

Recently, artificial intelligence (AI) and deep learning (DL) methods have been extensively involved in radiotherapy workflow, such as dose prediction (4–7). The DL-based methods perform well in automatic feature extraction and mapping transformation (5, 8). The dose prediction model can make an end-to-end mapping transformation between patients’ anatomical and dose distribution information with organs-at-risk (OARs) constraints (9–12). Compared with using the conventional treatment planning system (TPS), using the DL model to generate predicted dose distribution reduces planning time significantly (13–16).

Tomotherapy is a superior RT modality for treating advanced cancers, such as head and neck cancer. Compared to conventional RT treatment, Tomotherapy plan optimization is a time-consuming process. To make a plan with desirable quality, the planner needs to adjust the dose-volume histogram (DVH) limitation and plan criteria to update the plan weights iteratively. In this context, the total voxel information becomes a crucial consideration in dose prediction. It can guide Tomotherapy plan optimization, reducing the iteration times by lessening TPS optimization’s adjustment steps and minimizing the planning outcome uncertainty caused by anthropogenic factors. Different planners may handle the planning process.

Due to the complex anatomy, it is highly challenging to make a plan that can precisely deliver the prescribed dose to the target for the head and neck cancer patients (17, 18). They carry great essential functions for humans, and they need to be protected from unnecessary doses to guarantee which could still function well after the treatment (safe during the treatment). It results in more difficulty in achieving the desirable dose for planning target volumes (PTVs).

This study aims to establish the underlying relationship between anatomical and dose distribution information for nasopharyngeal carcinoma (NPC) patients treated with Tomotherapy using deep-learning approaches. Since few studies have been performed to investigate dose prediction for NPC, this study should be potentially exciting and valuable as guidance or reference for future RT planning.

## Materials and Methods

### Data Collection and Preparation

One hundred twenty-four NPC patients were treated with Tomotherapy, and our study collected their data. PTVs, the OARs, and the external contour (Body) were labeled as the contoured structures. We added a 3 mm margin around the gross tumor volume of the nasopharynx (GTVnx) and clinical target volume (CTV) to create the planning GTVnx (pGTVnx) and PTV, respectively. The PTVs include PTV_60_ (a prescription dose of 60 Gy) and PTV_54_ (54 Gy). The OARs included Brainstem, Spinal-cord, Eyes, Lens, Larynx-esophagus-trachea (L-E-T), Optic-nerves, Oral-cavity, Parotid-glands (PGs), Pituitary, Thyroid, Submaxillary-glands (SMGs). The study collected Digital Imaging and Communications in Medicine (DICOM) files for each case, including CT series, RT Plan, RT Structure, and RT Dose files. All cases corresponding DICOM files involved in our study have been done for particular quality assurance (QA) and delivered.

The collected cases have good consistency: have all PTV_60_ (with prescription dose of 60 Gy) and have the same types of OARs. We did the data preprocessing before the model training. It ensures the CNN network could load and correctly process the mapping transformation between the patient’s anatomical and dose distribution information. We extracted the 3D CT matrix from CT DICOM files, and the voxel values were normalized for each case. The normalized CT matrix holds a zero mean value and one as the variance. The study converted the region of interest (ROI) information to a binary mask, which means the pixels inside the contouring area with a value of 1 and pixels outside the contouring area with 0. The spacing and matrix shapes of the ROI contouring mask were adjusted equal to the corresponding CT matrix. We obtained the dose array from RT Dose files, with dose values (from 0 to 74 Gy) directly recorded in the dose matrix. All data preprocessing had been done by Python codes. NumPy, pydicom, and other python packages were used to conserve the raw data to the “npy” format.

### 3D Neural Network

**Figure 1:**
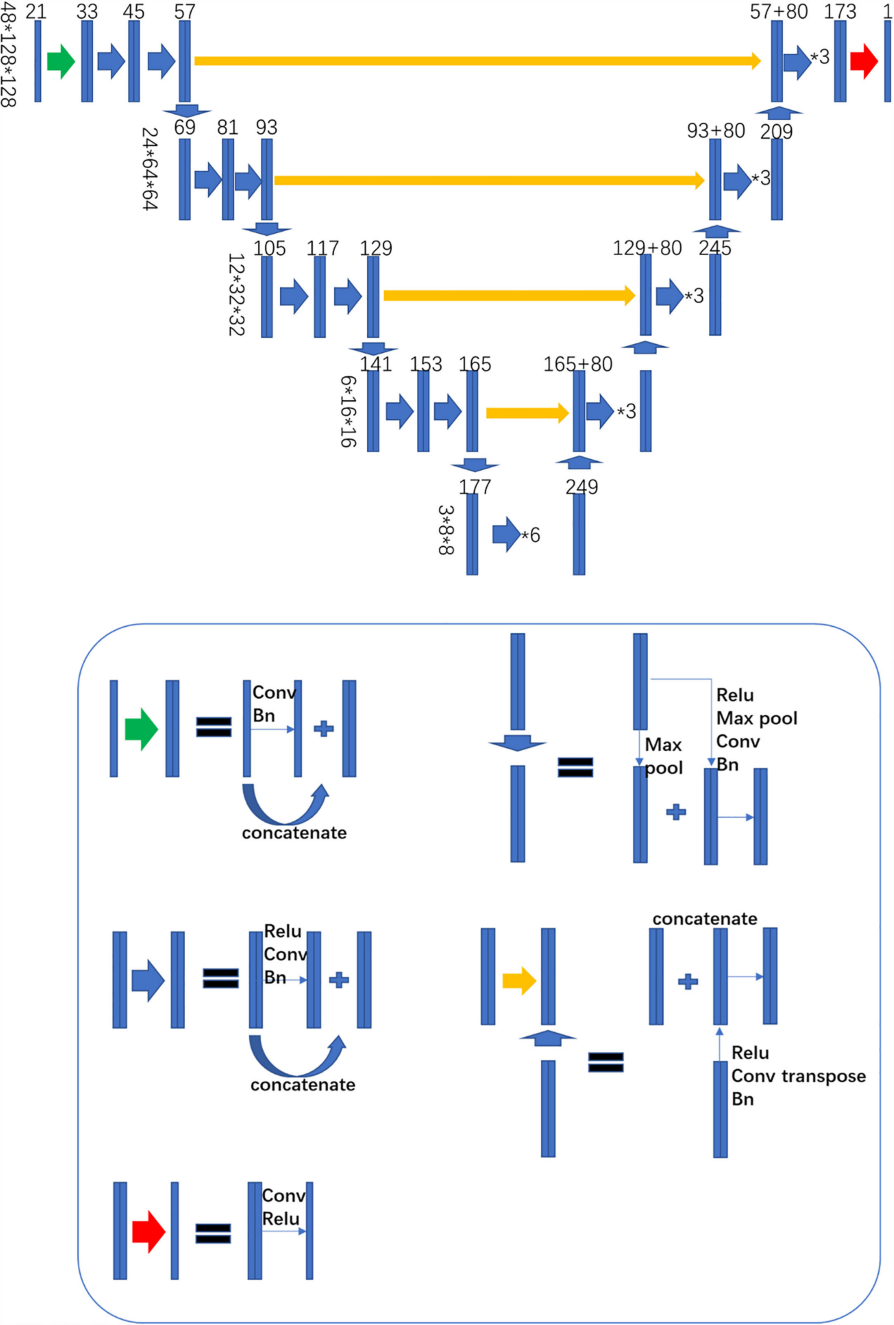
3D Dense-U-NET structure.

The 3D Dense-U-Net was built as the neural network architecture (**Figure 1**). “U-Net” is a famous well-behaved CNN network specializing in end-to-end matrix mapping (19). The U-Net architecture consists of down-sampling and up-sampling blocks concatenated across the bottleneck symmetrically, thus allowing the model to extract features for high, middle, and low level (20). The Dense-U-Net structure preserves the up-sampling and down-sampling portions and adds the densely connected layers within each hierarchical level to create the “Dense structure” (21). Every hierarchical level of Dense-U-Net preserves all features from previous layers. It allows the features to be reused and propagated along with successive layers. The 3D Dense-U-Net is the 3D version of the Dense U-Net model. Compared to the 2D Dense U-Net, the 3D one can directly process the input 3D matrix’s information and capture features along the Y direction.

### Training and Testing

For the 124 nasopharyngeal carcinoma cases, we randomly chose 96 cases for training and 28 cases for testing. The model input matrix contained 21 channels. The first channel is for CT image information, and the 2–21 channels contain ROIs contour information, which includes pGTVnx, PTV_60_, PTV_54_, Body, Brainstem, Spinal-cord, Eye-L, Eye-R, Lens-L, Lens-R, L-E-T, Optic-nerve-L, Optic-nerve-R, Oral-cavity, Parotid-L, Parotid-R, Pituitary, Thyroid, SMG-L, and SMG-R. In this study, the ground truth is the dose distribution from the collected RT DOSE DICOM files. Due to the GPU memory limitation, we specified the patch-training strategy. The 128×128×48 shape matrix for training was randomly selected from the 3D dose matrix. The 3D Dense-U-Net model was built up by connection of Dense Block. Every Dense Block includes a Relu activation process, followed by convolution (kernel size 3×3×3), batch normalization, and concatenation with the previous layer. We used zero paddings in each convolution, and each convolution layer had 12 channels. The 3D Dense-U-NET model went through four times down-sampled by max-pooling (kernel size 2×2×2) and symmetrically with four times up-sampled by deconvolution (kernel size 2×2×2, channel =80). The down-sampling process reduced the initial input matrix size from 128×128×48(128➔64➔32➔16➔8) to 8×8×3. It allows the network to be able to extract features both locally and globally; the up-sampling restored the matrix size from 8×8×3 to 128×128×48. The final hierarchical layer of convolution forms a single channel matrix and becomes the output matrix. We used the Adam optimizer (22) with the MAE loss function $\left(\frac{1}{n}\Sigma_{i=1}^{n}\left|f(x)-y\right|\right)$ and settled the batch size as 4. The learning rate decayed from 10^−4^ to 10^−6^ during CNN network training. When the loss values and learning rate stabilized, the process stopped training. And an Nvidia RTX 3090 GPU accelerated the entire training and testing process in this study. The deep learning framework was TensorFlow and Keras.

**Figure 2:**
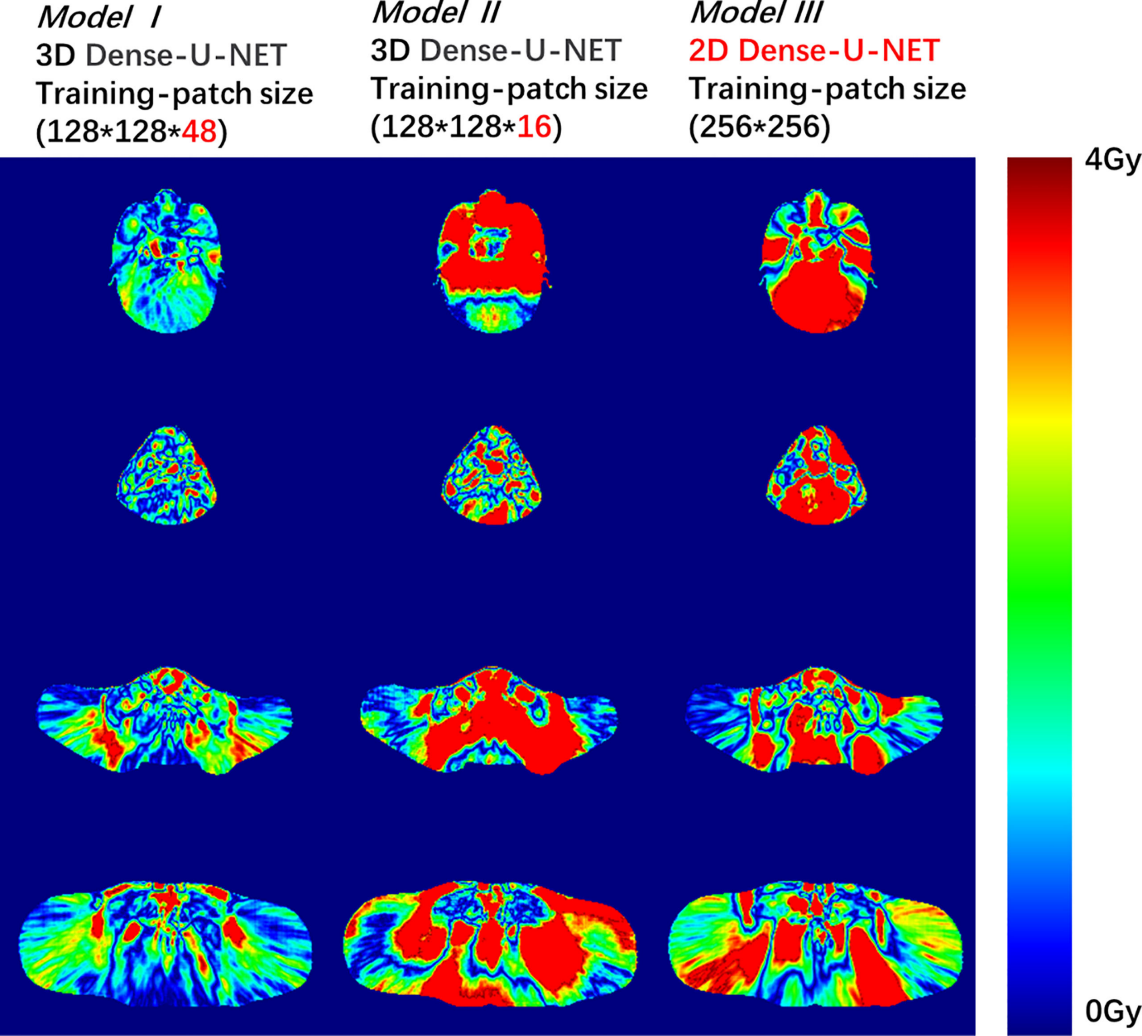
Dose difference between the predicted dose distribution and the ground truth for Model I, II, or III. The deep red color shows the dose difference beyond 4 Gy.

This study used 28 untrained cases for the model testing. The CT images and ROI contours were used as the model input data, and dose distribution was the model output (**Figure 2**). The matrix height (Y direction) of the testing case patch was 64. We concatenated the full-body dose distribution after the model generated the predicted dose distribution for each testing case patch.

We trained two comparative models with different Y lengths (height) to verify whether the 3D model with a large-height training patch could extract more interrelation information from different OAR-PTV distances in the Y direction. From our statistics results, the distance between specific OARs to PTV varies a lot among different patients. For example, the optic nerves’ distances to PTV ranged from 0 to 30 mm, which already equals 10 slices thickness of a CT scan with 3 mm thickness. Model I used the above model training method, and the shape of the training matrix was 128×128×48. Model II reduced the height of the training matrix to 128×128×16 shape. Training Model II aimed to verify whether the increase of height of the training matrix would be helpful to modulate the model to provide more accurate dose prediction for OARs. If the maximum distance from the optic nerve to PTV was 10 slices, and the height of the training matrix was just 16 slice distances, the training matrix may not be able to find enough spatial relationship from optic-organ to PTV. Increasing the height of the training matrix may allow the model to explore a more spatial relationship between the optic nerves and PTV, therefore, to generate more accurate OAR dose prediction. Model III used 2D Dense U-net. It is a simplified change from 3D Dense-U-net to a 2D version. Model III was designed to eliminate the “Y-direction distance” influence in the learning process. Model III can be seen as a comparison experiment to verify if the OAR-PTV distance in the Y direction would be a factor affecting the DL model output.

### Quantitative Evaluation

Percentage of errors (*δDi*), *p*-value, and gamma passing rate were calculated to evaluate our three models’ accuracy. The formula of the percentage of errors was:


$$\delta Di=\frac{\left|Di_{Ground-truth}-Di_{predicted}\right|}{prescription\;dose}$$

We calculated *δDi* of D_98_, D_95_, D_50_, D_2_ for PTV_60_, and D_mean_, D_max_ for all ROIs. All corresponding *δDi* for 28 test patients were counted and formed mean and standard deviation (Mean ± SD) for each ROI. The *p*-value of the two models’ *δDi* was calculated using a T-test; when the *p*-value<0.05, the prediction results have no statistical correlation. The gamma passing rates with the 3%/3 mm criteria and 10% threshold for the three approaches were calculated by 3D Slicer 4.10.2 [National Institutes of Health (NIH), USA] software.

## Results

The mean deviations from the mean and maximum dose of PTVs and OARs were 2.42 and 2.93%, respectively. Error for the maximum dose of optic nerves-R in Model I was 4.87 ± 6.88%, compared with 7.9 ± 6.8% in Model II (*p*=0.08) and 13.85 ± 10.97% in Model III (*p*<0.01); Model I showed well. The gamma passing rate of PTV_60_ for 3%/3 mm criteria was 83.6 ± 5.2% in Model I, compared with 75.9 ± 5.5% in Model II (*p*<0.001) and 77.2 ± 7.3% in Model III (*p*<0.01); Model I also did the best job. The prediction error of D_95_ for PTV_60_ was 0.64 ± 0.68% in Model I, compared with 2.04 ± 1.38% in Model II (*p*<0.01) and 1.05 ± 0.96% in Model III (*p*=0.01); Model I still performed well. The details of prediction errors are presented in **Table 1** and **Table 2**.

**Table 1:** Mean and standard deviation (Mean ± SD) of maximum and mean values between the predicted dose and the ground truth received on PTVs and OARs relative to the prescription dose.

ROI	Error of D_mean_ (%)					Error of D_max_ (%)				
	Model I	Model II	Model III	p^a^*	p^b^*	Model I	Model II	Model III	p^a^*	p^b^*
Body	0.58 ± 0.49	1.70 ± 1.17	0.64 ± 0.56	<0.01	0.67	2.09 ± 1.19	1.91 ± 1.07	1.97 ± 1.18	0.55	0.69
Brainstem	3.36 ± 3.00	5.63 ± 4.03	4.54 ± 3.38	0.02	0.16	2.90 ± 2.49	4.85 ± 3.66	3.61 ± 3.23	0.02	0.35
Spinal-cord	3.18 ± 4.56	8.65 ± 3.83	3.49 ± 4.06	<0.01	0.78	2.83 ± 2.41	4.84 ± 2.61	3.26 ± 274	<0.01	0.52
Eye-L	1.35 ± 1.14	1.61 ± 1.78	2.87 ± 2.30	0.50	<0.01	4.40 ± 3.41	7.08 ± 5.60	7.09 ± 5.13	0.03	0.02
Eye-R	1.48 ± 1.59	2.59 ± 2.92	2.31 ± 2.29	0.07	0.11	3.64 ± 3.31	9.89 ± 7.12	4.36 ± 495	<0.01	0.51
Lens-L	0.39 ± 0.34	0.48 ± 0.39	0.80 ± 0.61	0.31	<0.01	0.80 ± 0.66	0.80 ± 0.55	1.23 ± 0.96	0.99	0.05
Lens-R	0.52 ± 0.42	0.44 ± 0.35	0.63 ± 0.60	0.47	0.39	0.75 ± 0.59	0.69 ± 0.43	0.83 ± 0.77	0.67	0.66
L-E-T	2.20 ± 2.05	9.23 ± 3.92	2.73 ± 2.50	<0.01	0.38	2.49 ± 1.59	3.85 ± 3.61	2.28 ± 2.07	0.06	0.67
Optic-nerve-L	5.10 ± 4.40	7.67 ± 5.02	11.47 ± 9.38	0.04	<0.01	5.84 ± 5.21	8.30 ± 5.11	13.15 ± 11.58	0.07	<0.01
Optic-nerve-R	4.71 ± 5.12	7.90 ± 5.77	11.06 ± 8.11	0.03	<0.01	4.87 ± 6.88	7.93 ± 6.80	13.85 ± 10.97	0.08	<0.01
Oral-cavity	2.40 ± 2.20	2.63 ± 2.47	2.43 ± 2.13	0.70	0.96	2.12 ± 1.94	2.32 ± 1.95	2.21 ± 2.22	0.69	0.87
Parotid-L	2.13 ± 1.57	3.70 ± 2.60	1.60 ± 1.43	0.01	0.17	3.41 ± 2.62	3.90 ± 2.93	2.83 ± 2.08	0.50	0.35
Parotid-R	2.84 ± 2.29	4.00 ± 2.65	2.47 ± 2.29	0.08	0.53	3.46 ± 2.58	3.34 ± 3.03	3.37 ± 2.48	0.87	0.89
pGTVnx	0.56 ± 0.33	0.98 ± 0.65	0.68 ± 0.42	<0.01	0.24	1.92 ± 1.15	1.91 ± 0.86	1.81 ± 1.07	0.97	0.69
Pituitary	4.01 ± 5.36	4.23 ± 4.96	10.06 ± 9.59	0.87	<0.01	3.87 ± 4.17	4.47 ± 3.81	7.19 ± 8.76	0.57	0.07
PTV1	0.80 ± 0.49	1.27 ± 0.69	0.73 ± 0.77	<0.01	0.71	2.09 ± 1.19	1.91 ± 1.07	1.97 ± 1.18	0.55	0.69
PTV2	0.54 ± 0.54	1.29 ± 0.96	0.45 ± 0.32	<0.01	0.46	2.82 ± 2.01	2.49 ± 2.03	4.28 ± 3.33	0.53	0.04
Thyroid	3.66 ± 3.26	7.53 ± 4.16	4.59 ± 3.54	<0.01	0.29	2.23 ± 1.61	3.76 ± 2.78	2.01 ± 1.83	0.01	0.61
Mandible-L	3.77 ± 5.11	6.32 ± 5.94	6.21 ± 5.97	0.08	0.09	3.20 ± 2.67	5.09 ± 3.71	3.76 ± 3.20	0.03	0.47
Mandible-R	2.43 ± 1.97	3.23 ± 3.23	3.19 ± 4.13	0.26	0.37	2.71 ± 2.06	2.96 ± 2.63	2.09 ± 209	0.69	0.25

*: p_a_, p value between Model I and Model II; p_b_, p value between Model I and Model III.

**Table 2:** Means and standard deviations (Mean ± SD) of absolute differences for clinical DVH metrics between the predicted and ground truth doses.

	PTV_60_ error (%)				
	Model I	Model II	Model III	p_a_*	p_b_*
D_98_	1.24 ± 1.52	3.20 ± 2.18	1.19 ± 1.44	<0.01	0.68
D_95_	0.64 ± 0.68	2.04 ± 1.38	1.05 ± 0.96	<0.01	0.01
D_50_	1.07 ± 1.04	1.14 ± 1.31	0.93 ± 1.18	0.13	<0.01
D_2_	1.37 ± 0.80	1.52 ± 1.00	0.94 ± 0.89	0.65	<0.01

*: p_a_, p value between Model I and Model II; p_b_, p value between Model I and Model.

**Figure 3:**
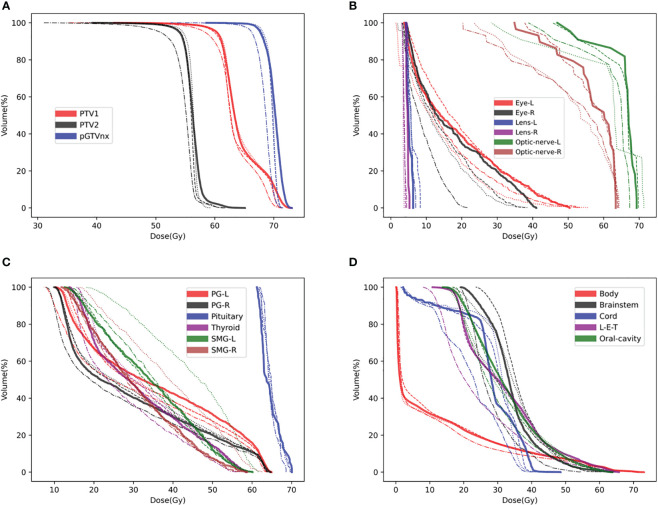
DVH plots for a test patient, such as **(A)** DVH plot of PTVs, **(B)** DVH plot of optic organs, **(C, D)** DVH plots of the other OARs. DVH, Ground-truth (Solid line), Model I (Dashed line), Model II (Dashed and dotted line), Model III (Dotted line).

To compare the three models’ accuracy intuitively, we randomly selected a test patient. We showed the dose difference between the predicted dose and the ground truth in **Figure 2** and the DVH plots of ROIs in **Figure 3**. Figures of the dose differences and DVH plots showed that Model I has the best prediction among the three models and an advantage in predicting the optic organs’ dose.

## Discussion

Precise automatic dose prediction can significantly improve clinical planning efficiency and safety (23). 3D dose prediction results can refer to current RT plan optimization in TPS (24, 25). Here, we built CNN-based dose prediction on the previous approved delivered plans. Since, in daily clinical practice, different medical physicists handled the planning process, which provided a source of uncertainty of the RT planning outcome. Using CNN-based dose prediction results guiding plan optimization can reduce the uncertainty of the planning outcomes and improve the plan optimization speed (26). A few fluence-prediction-based auto-planning researches have been done in the past few years. They mentioned that dose distribution could be predicted utilizing a fluence map as well. Furthermore, this enlightens us to get the dose prediction based on an auto-planning system (27, 28). Dose prediction studies can be the basis for much RT-relevant research and technology development.

NPC cases with Tomotherapy have great value in deep-learning dose prediction research. As we know, NPC patients with Tomotherapy are relatively rare in clinical RT practice. And in the past, studies about dose prediction of NPC patients with Tomotherapy were also not too many. Our study for dose prediction found that using a 3D CNN network for training could provide a better outcome than using a 2D CNN network, and the dose prediction accuracy has reached the clinical standard (the mean deviations for the mean and maximum doses of PTVs and OARs were 2.42 and 2.93%, respectively). It can refer to future dose prediction of NPC patients with Tomotherapy, even though this method still needs more research to improve its accuracy.

Our dose prediction model performed well in OARs and PTV areas but didn’t work well in the outside area of OARs and PTVs. Although the outcome accuracy in this study met the clinical requirements, the evaluating indicators included the deviations for the mean and maximum doses for ROIs, the gamma passing rates for PTV, and the DVH plots. But there are still some problems, such as the passing rate for Body was 70.2 ± 9.8%, which was relatively poor. That means further research should focus on how to predict accurate doses in no-contoured areas. Future studies recommend inputting more features such as the help region and the outward expansion area or controlling training data’s consistency, such as only using the designed plan from the same planner.

Besides building a dose prediction model, there is another critical factor that needs to be thought over (1). Training with the CNN network should follow the clinical logic concept (2). Training strategy should not directly duplicate from other studies, considering the dataset’s features should be ahead of training.

In this study, the network structure is similar to some medical imaging segmentation networks. Previous studies showed that the U-net could perform very well in dose prediction and CT image segmentation tasks. But the training strategy should be suitable for the specific prediction tasks. For example, the 2D U-Net can perform pretty well in the task of CT image segmentation (19, 29, 30). Slice by slice segmentation prediction is similar to the clinical logic flow. As is well known, clinical staff always creates the contouring slice by slice, which affirms that each single CT slice should contain enough segmentation information. But as shown in the study, directly using the 2D network to predict dose distribution slice by slice cannot give us the wanted outcome, which may be due to the loss of Y-direction information as shown in the results, the OARs (such as Spinal-cord), which were close to PTV in the Y direction. It didn’t show the different results using the 2D or 3D network to predict the dose distribution. But for OARs far from PTV in the Y direction, such as the optic organs, the dose-prediction results of the 2D network brought out lacks ability. The reason for the outcome difference could be that the algorithm logic is different from the clinical logic. When a medical physicist or dosimetrist makes a treatment plan, the staff should consider the relationship of the relative location between OAR and PTVs. We can quickly understand that it is difficult to avoid unnecessary doses for NPC patients if the optic organs are close to PTV. On the other hand, if the organs are far from PTV, they would be protected from radiation more efficiently. So, using the 3D network for training can allow the model to get the relative location between OARs and PTV. This action conforms to the clinical logic flow. Thus, a good outcome could meet.

Meanwhile, it is necessary to formulate the training strategy by considering the dataset’s features. Some deep-learning-based dose prediction studies have been made for cervical carcinoma. The studies used a general 3D-model-patch-training strategy with 16 pixels height matrix to train (shape of n×n×16) or directly used a 2D network for data training (5, 31, 32). From some dose prediction studies proposed, 2D network training is good enough to provide excellent results of dose prediction. But in transplanting the patch-training strategy to this project, using (n×n×16) shape matrix to train or using the 2D network gives different results. We found that the results were not so good. Reviewing the patient’s anatomic structure, we finally uncovered the dependency between the dose prediction results and the patient’s anatomic information. Using an (n×n×16) shape training matrix/patch, we got an ideal dose prediction for the patients whose PTV was close to the eyes. But for the patients whose PTV was far from the eyes, it resulted in a wrong prediction. The statistics results showed that the optic nerve’s dose delivered was negatively correlated with the distance from PTV to it. For all patients involved in the study, the maximum dose for optic nerves ranged from 9.7 to 71.4 Gy; the distance from the optic nerves to PTV ranged from 0 to 30 mm. The deep-learning model needs to know the spatial relationship between OARs and PTV. Since the predicted doses of optic nerves were highly related to its distance to PTV, using (n×n×16) shape matrix for training, it wouldn’t get an accurate dose prediction for the cases with sizeable PTV-eye distance. We believe that the (n×n×16) shape training matrix fitted better to extract anatomic information in pelvic cancer because the pelvic tissues were generally compact to PTV. For NPC patients, the PTV-eye distance varies from 0 to several centimeters. If the training patch’s height is small such as 16 pixels, it may be difficult for the deep-learning network to find the PTV-eye spatial relationship. PTVs have usually more than 70 slices thickness height for NPC patients. Suppose the training patch’s matrix with a considerable height, such as height = 48 pixels, and the model could extract more features of the spatial relationship among the PTV-eye voxels.

Clinical and actual treatment logic concept includes a lot of information, which are greatly important. We could utilize them to optimize the deep-learning network performance relevant to the RT aspect. The training matrix should be considered the network’s field of view from which the model could find the transformation relationship. Training with the 3D Dense-U-NET could predict each pixel’s dose value by considering the full input matrix. Increasing the input matrix height (Y direction) would be a strategy realized the extraction combination features of model training and clinical logic concepts. Increasing the height of the input matrix (increases the local sense of field) can make the DL model find more spatial features and relationships correlated to PTV-OAR distance, which provides a more accurate outcome for dose prediction.

The deep-learning-based dose prediction method still has many problems that need to be solved. Firstly, previous research never focused on excavating the data’s internal features and comparing the data differences. The anatomical information holds tremendous differences among different patients. Secondly, we can’t directly use the previous researchers’ method for deep learning, for different tumor types and treatment techniques have specific dose prediction methods. According to the tumor type and treatment mode, developing a specific dose prediction method can be a better way to improve dose prediction efficiency and accuracy. Our research was focused on adding the clinical logic concept with the deep-learning method together. Therefore, we developed a more reasonable deep-learning model training strategy.

A deep-learning-based study focuses on the relevant software and hardware, the clinical logic concepts, and the collected data characteristic. Combining computer technology, clinical logic flow, and data characteristics would be an ideal pathway to develop an excellent-performance dose prediction model.

## Conclusions

In this study, we successfully developed an accurate dose prediction model using a 3D convolutional neural network. It proves well for NPC patients with Tomotherapy. It also tells that exploring the spatial features between OARs and PTV is necessary for dose prediction. We found that a 3D DL model with a larger Y-dimension training matrix increases the accuracy of dose prediction outcomes. With this extra consideration, our accuracy improvement method of dose prediction is good enough to be considered a milestone for the automatic planning process with Tomotherapy and other RT techniques. The predicted results could be used as a reference or guidance for systematic clinical RT planning.

## Data Availability Statement

The original contributions presented in the study are included in the article/supplementary material. Further inquiries can be directed to the corresponding authors.

## Ethics Statement

This study was approved by the Ethics Committee of the Chinese PLA General Hospital (approved no. S2016-122-01). Written informed consent to participate in this study was provided by the participant’s legal guardian.

## Author Contributions

YL: Experiment design and code implementation; article writing. ZC: Technical support. JW: Data collection. XW: Technical support. BQ: Data collection. LM: Data collection. WZ: Technical support, article modification. SX: Experiment design, article modification. GZ: Article modification. All authors contributed to the article and approved the submitted version.

## Funding

This work was supported by the Medical Big Data AI R&D Project (2019MBD-043).

## Conflict of Interest

Manteia Technologies Co., Ltd employed author ZC.

The remaining authors declare that the research was conducted in the absence of any commercial or financial relationships that could be construed as a potential conflict of interest.

## Publisher’s Note

All claims expressed in this article are solely those of the authors and do not necessarily represent those of their affiliated organizations, or those of the publisher, the editors and the reviewers. Any product that may be evaluated in this article, or claim that may be made by its manufacturer, is not guaranteed or endorsed by the publisher.
